# Non Steroidal Anti-Inflammatory Drugs and Inflammatory Bowel Disease

**DOI:** 10.3390/ph3041084

**Published:** 2010-04-12

**Authors:** Amir Klein, Rami Eliakim

**Affiliations:** Department of Gastroenterology, Rambam Health Care Campus, Haifa, P.O.B. 9602, Haifa, 31096, Israel

**Keywords:** NSAIDs, IBD, Crohn's, Ulcerative Colitis

## Abstract

Inflammatory Bowel Diseases (IBD) are an immune mediated chronic or relapsing disorders of the gastrointestinal (GI) tract. IBD is characterized by a chronic intestinal inflammatory process with various components contributing to the pathogenesis of the disease including environmental factors such as smoking or use of Non Steroidal Anti-Inflammatory Drugs (NSAIDS). NSAIDS are among the most commonly used medications for the treatment of various inflammatory conditions. The main factor limiting NSAIDS use is the concern for the development of gastrointestinal toxicity including mucosal injury. A possible association between the use of NSAIDS and the onset or relapse of IBD has been repeatedly suggested. This article will review the current concepts and evidence of the relationship between IBD and NSAIDS.

## 1. Introduction

Inflammatory Bowel Diseases (IBD) are an immune mediated chronic or relapsing disorders of the gastrointestinal (GI) tract and consist mainly of Crohn's disease (CD) and Ulcerative colitis (UC). IBD is characterized by a chronic intestinal inflammatory process with various components contributing to the pathogenesis of the disease including environmental factors (such as smoking or NSAIDS), genetic background, host intestinal flora and the host immune system [1].

CD is a transmural disease characterized macroscopically by skipped lesions with aphtae, ulcers of various sorts and at times the classical cobble-stone appearance. Microscopically a trans-mural chronic inflammation is present with or without granuloma formation. The chronic inflammation of UC is confined to the mucosa and is continuous in nature. Macroscopically a granular appearance or an edematous hemorrhagic mucosa, in more severe cases, is typical. Microscopically crypt architectural distortion and chronic inflammation are typical [1].

Since CD can affect any part of the GI tract, the clinical spectrum of the disease varies widely from epigastric pain and helicobacter pylori negative gastritis, due to upper GI involvement, to diarrhea, weight loss, abdominal pain and nutritional deficiencies in patient with small bowel disease. Hematochezia, bloody diarrhea, tenesmus and occasional fever are indicative of colonic involvement in CD or the presence of UC. Peri-anal disease is confined to patients with CD. 

Up to a third of IBD patients suffer from extra intestinal manifestations of their disease. These include dermatologic manifestations such as erythema nodosum, ocular complications such as conjunctivitis and uveitis, hepatobiliary complications including cholelithiasis, steatosis and primary sclerosing cholangitis and urologic complications mainly nephrolitiasis. Finally, two very important and prevalent extra intestinal complications are rheumatic manifestations such as arthralgias, peripheral arthritis or ankylosing spondilitis and osteoporosis with increased risk of fractures secondary to vitamin D and calcium deficiency and prolonged steroid use [2,3]. For both these complications, and for non IBD related pain, patients with IBD often seek relief in NSAIDS [4,5].

Non Steroidal Anti-Inflammatory Drugs are among the most commonly used medications for the treatment of various inflammatory conditions. An estimated 60 million Americans use NSAIDS regularly [6]. The main factor limiting NSAIDS use is the concern for the development of gastrointestinal toxicity including mucosal injury in the form of erosions and ulcers, upper GI, small bowel or colonic bleeding and rarely perforation and obstruction due to stricture formation [7]. NSAIDS may also cause a non specific type of colitis and small intestinal inflammation with associated complications of chronic blood or protein loss [8]. Endoscopic features of NSAIDS induced colonic damage include sharply demarcated or circumferential ulcers which are usually reversible upon discontinuation of the drug [9]. It is estimated that NSAIDS use by patients with arthritis in the US causes over 100,000 hospitalizations annually for GI complications, one fifth of which are estimated to be due to lower GI complications [10]. A review of the literature on lower GI adverse effects of NSAIDS revealed that there was a statistically significant increase in adverse outcome rates associated with the use of NSAIDS in patients with lower GI bleeding, perforation and complicated diverticular disease [10]. A possible association between the use of NSAIDS and the onset or relapse of IBD has been repeatedly suggested. However, lack of controlled prospective trials, make it difficult to draw definite conclusions [4,11].

## 2. Pathogenesis of NSAIDS Induced GI Toxicity

Several mechanisms responsible for NSAIDS induced GI toxicity in general and in IBD patients specifically have been suggested. These include, increased mucosal permeability, formation of drug-enterocyte adducts, and NSAIDS induced intracellular ATP deficiency and increased enterohepatic circulation. However, the most discussed mechanism is their effect on prostaglandin synthesis [4]. Prostaglandins have a pivotal role in mucosal defense, maintenance of microcirculation and modulation of the immune system in the colon [12]. Experimental models using active immunization against prostaglandins E2, F2A and D2 and inhibition of COX1 and COX2 resulted in the development of intestinal ulcers and exacerbation of dextran sulfate sodium (DSS) induced colitis [13,14]. Reduced prostaglandin production due to inhibition of COX1 and COX2, has been implicated in the early and frequent clinical relapse of IBD [15].

COX2 is an inducible enzyme not detected in normal epithelium. Its expression is induced within a few hours of exposure to invasive organisms or pro-inflammatory cytokines including interleukin 1 and tumor necrosis factor alpha and is usually lost within 24 hours. Many of these events involve the NFK-B signaling pathway [16]. In areas of active inflammation due to IBD, COX2 enzyme can be detected in apical epithelial cells of the inflamed mucosa [4,17]. The expression of COX2 in the small intestinal and colonic mucosa was found to be higher in experimental colitis, to correlate with inflammatory activity in IBD and to have a beneficial effect on healing in experimental colitis [18,19]. The exact mechanism by which COX2 inhibitors induced relapse in IBD is uncertain. Several hypotheses have been suggested including delayed wound healing with increased vascular permeability, maintenance of intestinal mucosa integrity and anti-inflammatory properties in intestinal tissue during inflammation [4,20].

**Figure 1:**
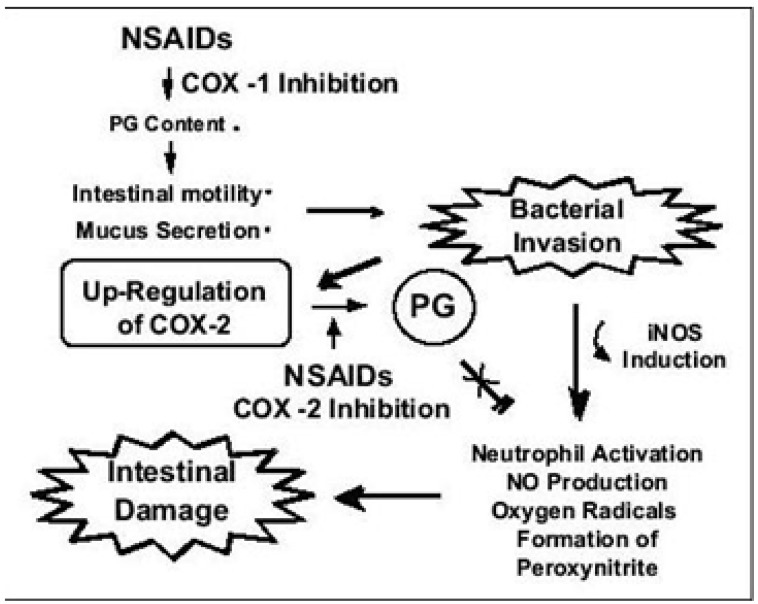
The potential pathogenesis of NSAIDS induced GI toxicity [36]. Adapted with permission from the publisher.

**Table 1:** Possible molecular mechanisms of NSAIDS induced IBD exacerbations [4]. Adapted with kind permission from Springer Science+Business Media.

Mechanism of action
**Prostaglandin synthesis reduction - Reduced COX1 and COX2 induced PGE2 and TXA2 production - Immunomodulatory and anti inflammatory role in the GI tract. ** **Mucus phospholipid membrane interaction** **Effect on mitochondrial energy metabolism – ATP deficiency and increased mucosal permeability** **Increased enterohepatic circulation** **Formation of drug-enterocyte adducts** **COX independent GI toxicity** **Increased TNF-α, IL-10 and NO release** √ **Impaired mucosal microcirculatory blood flow ** √ **Impaired mucus secretion and acid regulation ** √ **Impaired renal blood flow ** ○ **Loss of vasodilatation** ○ **Increased vascular permeability** ○ **Delayed wound healing** ○ **Increased leukocyte adherence to vascular epithelium** ○ **Increase reactive oxygen metabolites**

√ Mechanism attributed mainly to COX1 inhibition. ○ Mechanism attributed mainly to COX2 inhibition.

## 3. Conventional NSAIDS and IBD

NSAIDS may initiate IBD, or cause reactivation of quiescent disease and induce GI complications [15,21,22]. There is a higher than expected rate of NSAIDS use among patients admitted to the hospital for IBD flares [22]. Several retrospective and cohort studies have implicated NSAIDS in the onset or exacerbation of IBD [23,24,25]. Takeuchi *et al.* [15], using clinical evaluation and fecal calprotectin measurements demonstrated that non-selective NSAIDS were associated with a 17–28% relapse rate within nine days of ingestion of the drug, in patients with IBD. Meyer *et al.* [24], retrospectively reviewed files of outpatient with IBD and showed that treatment with NSAIDS was associated with disease relapse. Several additional case control studies have shown that NSAIDS increase the risk of new onset of IBD [26,27], subsequent flares [25,27] and are associated with a higher disease activity index [28]. In contrast, two studies by Bonner *et al.* [27,28] showed no association between either active or quiescent CD or UC and the use of NSAIDS in a population of outpatients with IBD. 

## 4. Selective COX2 Inhibitors and IBD

Selective COX2 inhibitors cause less GI toxicity compared to conventional NSAIDS. To date, studies on the use of selective COX2 inhibitors in IBD have yielded mixed results. Mahadevan *et al.* [29] evaluated 27 patients with CD, UC and pouchitis receiving Rofecoxib or Celecoxib. Treatment was shown to be both beneficial and safe. A large double blind placebo controlled trial by Sandborn *et al.* [30] of 222 UC patients in remission with arthritis or arthralgias demonstrated that up to two weeks treatment with Celecoxib did not result in a higher relapse rate than placebo. Another multicenter double blind placebo controlled trail by Miedeny *et al.* [31] included 146 patients with IBD receiving either Etoricoxib or placebo for three months. Treatment was beneficial and safe and was not associated with disease flare. Another open label trial by Reinisch [32] demonstrated the efficacy and safety profile of Rofecoxib in similar patients.

**Table 2:** Articles on the effect of NSAIDS and selective COX2 inhibitors on IBD.

Article	Type of study	Type of IBD	Type of NSAIDS	Conclusions
**Takeuchi, K. *et al.* [15]**	Prospective cohort	UC and CD	Non selective	NSAIDS ingestion is associated with frequent and early relapse of quiescent IBD.
**Meyer A.M. *et al.* [24]**	Retrospective cohort	UC and CD	Non selective	Use of NSAIDS was associated with relapse of IBD.
**Felder J.B. *et al.* [25]**	Case control	UC and CD	Non selective	NSAIDS provoke disease activity in both UC and CD.
**Evans, J.M. *et al.* [26]**	Case control	UC and CD	Non selective	NSAIDS are associated with hospitalizations for severe colitis in patient with IBD.
**Bonner, G.F. *et al.* [27]**	Retrospective cohort	UC and CD	Non selective	NSAIDS use was not associated with higher likelihood of active IBD.
**Bonner, G.F. *et al.* [28]**	Case control	UC and CD	Non selective	High dose NSAIDS were associated with higher disease activity index but no significant disease flares were observed.
**Mahadevan, U. *et al.* [29]**	Retrospective cohort	UC and CD	COX2 selective	COX2 inhibitors appear to be safe and beneficial in patients with IBD.
**Sandborn, W.J. *et al.* [30]**	Randomized placebo-controlled trial	UC	COX2 selective	Celecoxib treatment was not associated with greater relapse rates compared to placebo.
**El Miedany, Y. *et al.* [31]**	Randomized placebo-controlled trial	UC and CD	COX2 selective	Etoricoxib treatment was safe and beneficial in patients with IBD. It was not associated with IBD exacerbations.
**Reinisch, W. *et al.* [32]**	Prospective open label study	UC and CD	COX2 selective	Rofecoxib treatment was safe, beneficial and not associated with flares of IBD.
**Xin-Pu Miao *et al.* [35]**	Meta-analysis	UC and CD	COX2 selective	Insufficient data to determine the impact of COX2 inhibitors on IBD exacerbations.

In contrast, several case reports of exacerbations in patients with IBD receiving COX2 inhibitors have been reported [33,34]. Bioncone *et al.* [16] evaluated the safety and efficacy of COX2 inhibitors in an open label study. Rofecoxib controlled the arthralgias in two thirds of the patients, however side effects requiring discontinuation of the medication were observed in one fifth of the patients with IBD. These included abdominal pain, diarrhea, bloody stool and heart burn. Matuk *et al.* [32] evaluated the safety and toxicity of Celecoxib and Rofecoxib in 33 patients with IBD. All patients experienced a flare of their disease within 6 weeks of initiating COX2 therapy and 38% of them had resolution of their symptoms upon discontinuation of the treatment. Finally, a recent meta-analysis [35], on the use of COX2 inhibitors in IBD patients concluded that there is insufficient data to determine the impact of COX2 inhibitors on IBD exacerbations. These mixed finding suggest that further evaluation of the use of COX2 selective inhibitors in patients with IBD is required. Table 2 summarizes studies on the effect of NSAIDS on IBD.

## 5. Summary and Conclusions

In conclusion, to date, the available data in the literature on the effects of NSAIDS on IBD activity is inconclusive. It remains unclear whether NSAIDS and COX2 selective inhibitors indeed cause flares of IBD and whether COX2 inhibitors are safer than conventional NSAIDS. There is some evidence to suggest that short term usage of COX2 selective inhibitors is safe and beneficial in patients with IBD. Concomitant use of NSAIDS or COX2 inhibitors and steroids in patients with IBD warrants special consideration and careful monitoring due to the potentially increased cardiovascular and UGI toxicity, especially in older patients. Further research and controlled future clinical trials will help clear some of these topics. 
